# DGTTSSA: Data Gathering Technique Based on Trust and Sparrow Search Algorithm for WSNs

**DOI:** 10.3390/s23125433

**Published:** 2023-06-08

**Authors:** Walid Osamy, Ahmed M. Khedr, Bader Alwasel, Ahmed Salim

**Affiliations:** 1 Computer Science Department, Faculty of Computers and Artificial Intelligence, Benha University, Benha 13513, Egypt; walid.osamy@fci.bu.edu.eg; 2 Unit of Scientific Research, Applied College, Qassim University, Buraydah 52571, Saudi Arabia; 3 Computer Science Department, University of Sharjah, Sharjah 27272, United Arab Emirates; akhedr@sharjah.ac.ae; 4 Mathematics Department, Zagazig University, Zagazig 44523, Egypt; a.salem@qu.edu.sa; 5 Department of Computer Science, College of Science and Arts, Qassim University, P.O. Box 931, Buridah 51931, Saudi Arabia

**Keywords:** data collection, swarm intelligence, Internet of Things (IoT), trust, clustering, metaheuristic, Wireless Sensor Networks

## Abstract

Wireless Sensor Networks (WSNs) have been successfully utilized for developing various collaborative and intelligent applications that can provide comfortable and smart-economic life. This is because the majority of applications that employ WSNs for data sensing and monitoring purposes are in open practical environments, where security is often the first priority. In particular, the security and efficacy of WSNs are universal and inevitable issues. One of the most effective methods for increasing the lifetime of WSNs is clustering. In cluster-based WSNs, Cluster Heads (CHs) play a critical role; however, if the CHs are compromised, the gathered data loses its trustworthiness. Hence, trust-aware clustering techniques are crucial in a WSN to improve node-to-node communication as well as to enhance network security. In this work, a trust-enabled data-gathering technique based on the Sparrow Search Algorithm (SSA) for WSN-based applications, called DGTTSSA, is introduced. In DGTTSSA, the swarm-based SSA optimization algorithm is modified and adapted to develop a trust-aware CH selection method. A fitness function is created based on the nodes’ remaining energy and trust values in order to choose more efficient and trustworthy CHs. Moreover, predefined energy and trust threshold values are taken into account and are dynamically adjusted to accommodate the changes in the network. The proposed DGTTSSA and the state-of-the-art algorithms are evaluated in terms of the Stability and Instability Period, Reliability, CHs Average Trust Value, Average Residual Energy, and Network Lifetime. The simulation results indicate that DGTTSSA selects the most trustworthy nodes as CHs and offers a significantly longer network lifetime than previous efforts in the literature. Moreover, DGTTSSA improves the instability period compared to LEACH-TM, ETCHS, eeTMFGA, and E-LEACH up to 90%, 80%, 79%, 92%, respectively, when BS is located at the center, up to 84%, 71%, 47%, 73%, respectively, when BS is located at the corner, and up to 81%, 58%, 39%, 25%, respectively, when BS is located outside the network.

## 1. Introduction

In recent years, IoT has emerged as one of the most prominent areas of research where researchers hope to take control of daily activities through the Internet and improve the quality of life [1,2,3,4,5,6]. Various technological objects from the environment (e.g., cars and smartphones) are included in IoT [7,8]. With the integration of computational capabilities, it has become capable of achieving a huge leap in a variety of applications including home, military, healthcare, entertainment, and many other disciplines [9,10,11]. The Wireless Sensor Network (WSN) is the base technology underpinning the IoT, which has recently sped up advancements in several applications [12,13,14,15,16,17,18]. WSN can be comprehended as a contemporary information technology that integrates a variety of components, such as sensors, wireless communication, distributed data processing, and low-power embedded components, into a single system. In a WSN, there are many low-cost Sensor Nodes (SNs) that can detect, monitor, and gather environmental data in real time. These SNs can be static or dynamic. The self-organization and collaboration of WSNs have opened up a variety of real-time application possibilities including military, disaster relief, and emergency services that demand real-time information for effective planning and coordination [19]. Many applications, such as disaster recovery, video conferencing, and emergency treatment, have witnessed the considerable use of WSNs. The primary benefits of the WSN are its low price and distributed intelligence. It saves costs on installation and maintenance since it uses inexpensive wireless devices [20]. The IoT combines a number of technologies, including big data, WSN, and cloud computing [21,22]. Sensing, collecting, storing, and transmitting data to the Base Station (BS) are the main tasks of the IoT elements [23,24]. Data collection from large WSNs is a major obstacle for the development of IoT technology [25,26]. Therefore, it is required to find creative ways that extend the lifetime of the networks [27,28].

Clustering is a logical way of arranging SNs into different groups called clusters based on various pre-defined criteria that may include network load balancing, optimizing resource consumption, Quality of Service (QoS), etc., for optimal energy usage and an enhanced WSN lifetime. By electing an energy-aware CH, the network lifetime can be extended. Many research papers have investigated how the energy parameter affects CH selection, cluster construction, and data routing. Given the fact that the data is transferred over CHs, the level of security of the CHs must also be considered because of the possibility of malicious nodes which have a tendency to drop and reroute data, lowering data delivery rates and network accuracy. In addition to serving a crucial function, CHs are often the key targets of attackers, and once they are compromised, the cluster and its data become untrustworthy. A number of studies have worked on WSN security and malicious node identification by utilizing trust-based approaches due to the fact that the trust-based methods are found to be successful in ensuring WSN security [29]. A trust management strategy is an appropriate approach to identify faulty or malicious nodes in WSNs since it provides robustness to the network and ensures secure data delivery and resource sharing [30,31,32].

The significance of trust management in WSN can be illustrated by considering the scenario of electing a malicious node as a CH. All cluster members (CMs) that depend on that CH for packet delivery will indeed be negatively impacted. Malicious nodes will compromise the integrity of the data. In addition, the network’s overall performance depends heavily on determining the suitable CHs with enough remaining energy. Therefore, it is demanded that a clustering technique should consider the trustworthiness of nodes as well as energy consumption in order to achieve improved data security, efficiency, and reliability.

**Figure 1:**
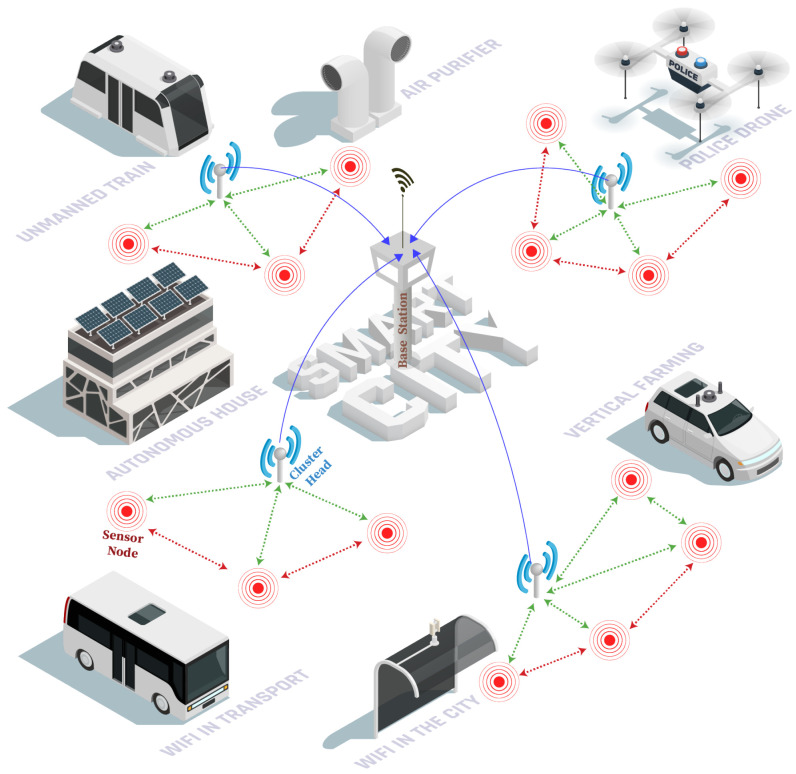
A general architecture of DGTTSSA.

The goal of this study is to create a trust-based clustering approach for WSN-based applications. This work proposes an energy-efficient and safe CH selection mechanism based on SSA for providing trust-enabled data gathering in WSNs. We develop a clustering technique with trust management that takes into account the energy usage and the trustworthiness of nodes in order to achieve improved data security since the overall performance of the network depends on selecting trustworthy CHs with sufficient residual energy. A general architecture of DGTTSSA is shown in Figure 1.

The main contributions of this work can be enumerated as follows:

1. We employ a new procedure that dynamically changes the trust and energy threshold values in order to be aware of the changes in the WSN during round operations.
2. We propose a new fitness function for CH election that takes into account remaining energy and trust values, weighted by the number of sensor nodes and adhering to threshold conditions.
3. For the efficient election of CH, the SSA, as one of the Swarm Intelligence optimization approaches, is modified and employed.
4. A comprehensive comparison between our proposed DGTTSSA approach with existing state-of-the-art clustering algorithms is provided.

The remainder of the paper is organized as follows: Section 2 provides the literature review of related algorithms, Section 3 gives the necessary preliminaries, and Section 4 details the proposed DGTTSSA. Section 5 presents the experimental results of DGTTSSA. Finally, Section 6 concludes the paper.

## 2. Related Work

Much research attention has been paid to the development of effective methods that improve the data-gathering efficiency of WSNs [33,34,35,36,37]. The routing method must satisfy diverse needs including QoS, minimal delay, security, and energy efficiency. In recent years, the use of trust-enabled data collection techniques has been recognized as a successful strategy for enhancing the security and data collection capabilities of WSNs.

For identifying the trustworthy nodes, the authors of [38] employed a random repeat method. The authors of [39] suggested a trust-based method (ETOR) with a hybrid objective function to assess the accuracy of nodes in distinguishing hostile nodes from normal nodes. The two primary parts of this algorithm are to choose reliable nodes with the help of a tolerance constant, and then to designate opportunistic nodes among the reliable nodes to perform routing. The authors of [40] provided a realistic trust-based routing approach to combat malicious nodes. The suggested method (TASRP) is a multi-factor routing approach that uses path length, residual energy, and node trust scores to generate energy-efficient routing paths between trustworthy nodes. The use of trustable nodes for data forwarding and reduced energy usage are both facilitated by this multi-factor technique. Another trust-based approach for WSNs using an adaptive GA, known as TAGA, is proposed in [41]. The method has been demonstrated to be effective in defending against special trust attacks and typical routing attacks. Furthermore, it minimizes energy consumption during data transmission in WSN.

Enhanced network lifetime requires both security and energy efficiency to be taken into account in WSN-based IoT designs. Energy limitations can be addressed by clustering the sensor nodes. It has become increasingly important to have trust in sensor data used for mission-critical IoT applications. A fuzzy-based trust aware method (TEEFCA) is proposed in [42] as an energy-efficient and secure clustering-based routing strategy for addressing these problems. To extend the WSN lifetime, which is an NP-hard issue, numerous meta-heuristic clustering techniques have been developed. An energy- and data-trust model is proposed in [43] for detecting untrusted nodes in clusters. Moreover, stochastic fractal search optimization is incorporated into the clustering protocol to optimize the WSN lifetime. Because the removal of the aggressor nodes has a striking effect on the network performance, an efficient routing through the node stability trust evaluation is proposed in [44]. The trust-based routing approach (TBSEER) in [45] addresses the issues of conventional trust management systems, which are only built to take into account one sort of assault and lack the capacity to swiftly detect untrusted nodes. The TBSEER creates a comprehensive trust value that is resistant to black holes, sinkholes, selective forward and hello flooding using adaptive trust (indirect and direct), and energy trust values.

To overcome the drawbacks of clustering techniques, such as a shortened CH lifetime, a WSN solution must consider the optimization of routing protocols, trust management, and CH selection strategies effectively. To this end, a Cuckoo search optimization algorithm is presented in [46] to enhance the confidence level and lifetime of the WSN. This algorithm uses type-2 fuzzy logic and clustering to achieve the optimal results. A unique hybrid approach to optimize the CH selection process is developed in [47] by taking into account the energy, delay, QoS, separating distance, and trust (indirect and direct). In [48], a Reliable CH selection method utilizing energy and trust-integrated semi-markov predictions with the goal of prolonging the lifespan of WSNs is proposed (RCHST-IETSMP). For an efficient CH election, the suggested RCHST-IETSMP integrated two key parameters related to trust and energy. Using deep learning and meta-heuristics, Ref. [49] adopted the trust-aware CH selection protocol in WSNs. This algorithm selects the optimal CH using a multi-objective function in terms of trust, delay, energy, and distance. For routing data packets in a secure path, a Particle-Water Wave Optimization (P-WWO) method is proposed in [50], which is an integration of PSO and WWO. To address the security issues in routing, Ref. [51] introduced a trust-enabled secure method enhancing LEACH (STELR). Emperor Penguin Optimization (EPO) is utilized to determine the CH for data aggregation. The LEACH, ACO, and ECC algorithms are integrated in [52] to determine the CHs and reduce cluster energy usage. TE-MHOA is proposed in [53] to improve the WSN lifetime by utilizing an energy and trust-aware multi-objective hybrid optimization algorithm.

Reputation and trust are heavily influenced by probability distribution functions. A trust model is developed in [54] for clustered routing in WSN. The calculation of trust values has been done using probability distribution functions and metaheuristic algorithms. By employing an efficacious clustering and CH decision process, Ref. [55] enhanced the energy efficiency as well as the network lifetime. In this process, CHs are chosen by examining the node residual energy and the distance to the BS. The protocol LEACH-TM proposed in [56] is a clustering protocol based on LEACH and relies on trust management. By taking into account the CH nodes count, remaining energy, and density of neighbor nodes, LEACH-TM enables a better energy load balance and transmission reliability among the nodes. The work in [57] minimized the possibility of a malicious node becoming the CH by creating an efficient and reliable trust mechanism. The overhead associated with calculating trust is also reduced. The study in [34] examined how battery power consumption was affected and lowered when normal nodes choose appropriate CHs with close proximity to the BS, and developed a method by improving LEACH for effective clustering. The results reveal an improved performance in extending the network’s lifetime. The work in [58] presented a trust-based security method based on Elephant Herd Optimization (EHO), which is a metaheuristic method that solves optimization problems based on elephant herding behaviors in clans. The suggested routing strategy chooses the best secure paths for data transmission based on trust values. In order to perform safe and energy-aware clustering, a hybrid Moth Flame Optimization (MFO) and GA-based strategy termed eeTMFOGA is introduced in [37]. eeTMFOGA uses the advantages of MFO to choose CHs among SNs. An objective function that considers average cluster distance, node density, remaining energy, and trust characteristics is used to select the CHs. In [59], the optimum CH selection and the secure path formation between the target nodes are achieved using a trust-aware method based on Multi-objective Black Widow Optimization (TC-MBWO). The CH selection and generation of a secure data transmission path are achieved using a number of factors, including the degree of nodes, distance, remaining energy, and trust. Moreover, the TC-MBWO takes trust into account to lessen hostile attempts during data transfer.

In contrast to the existing methods, the proposed DGTTSSA modifies and adapts the swarm-based SSA optimization algorithm to develop a trust-aware CH selection method. We employ a procedure that dynamically changes the trust and energy threshold values in order to be aware of the changes in the WSN during round operations. Based on the node’s trust value and residual energy, this work proposes a new energy-efficient and secure CH election mechanism for WSN-based applications.

A comparison of previous literature studies and the proposed DGTTSSA is given in Table 1.

## 3. Preliminaries

This section discusses the problem formulation, followed by the system model, energy model, and trust model used in the proposed work.

### 3.1. Problem Formulation

The clustering concept works by electing CHs among the SNs. These CHs then gather information from the CMs of their cluster, perform data aggregation, and then report the resultant data to the sink. However, being a CH could consume a considerable amount of energy. As a result, rotating the CH role could provide significant energy savings. The ability to control the energy distribution in a network in order to guarantee a long lifetime for the network is among the most crucial aspects for determining the efficacy of a clustering protocol for distributed WSNs. Designing a protocol capable of uniformly distributing energy within a network is not an easy task. On the other side, in cluster-based WSNs, security is an important and challenging topic. A WSN may have both malicious and normal nodes. The malicious (or compromised) nodes can act as CHs, thereby compromising the network performance. As a result, determining which SN is the most trusted by all CMs is, therefore, one of the most challenging aspects while selecting a CH.

This research aims to provide a trust-based CH selection approach for WSN that not only increases the WSN’s lifetime but also assures that the resources in the WSN are used effectively and efficiently by devising a protocol that can distribute energy consumption evenly across all nodes.

### 3.2. System Model

The development of the proposed framework is guided by the following considerations. We assume that *n* SNs are distributed uniformly in an area *R* of dimension A×A and a fixed BS. Assumptions used to build the network are as follows:

1. The SNs are capable of self-localization [60,61,62].
2. All SNs are static, and each SN has a unique ID.
3. SNs are assumed to have the ability to communicate and listen to their neighbors within their communication range for the purpose of trust evaluation.
4. An individual CH is responsible for each cluster management, which is awake during each round of operation.
5. CHs receive data from SNs about the environment (such as humidity or temperature).

### 3.3. Energy Model

SNs in a cluster are required to use energy for transmitting data, and each CH is required to use energy for data transmission, processing, and reception. In addition, the amount of energy required for data communication varies with distance. The energy model we used is similar to the one employed in a number of research works [33,34,35,36,63]. The expended radio power details for transmitting and receiving a message are given in the Equations (1) and (2), where the message size is l−bits and distance is *d*.

(1) $$E_{Tx}\left(l,d\right)=\left\{\begin{array}{cc} lE_{elec}+l\epsilon_{fs}d^{2} & \;\;\;\;\;d<d_{0}\\ lE_{elec}+l\epsilon_{mp}d^{4} & \;\;\;\;\;d\geq d_{0} \end{array}\right.$$

(2) $$E_{Rx}\left(l\right)=lE_{elec}$$

The parameters used for the simulated model are: $E_{elec}=50$ nJ/bit, $\epsilon_{fs}=10$ pJ/bit/m${}^{2}$, $\epsilon_{mp}=\frac{13}{1000}$ pJ/bit/m${}^{4}$, $d_{0}=\sqrt{\frac{\epsilon_{fs}}{\epsilon_{mp}}}$.

### 3.4. Trust Model

**Figure 2:**
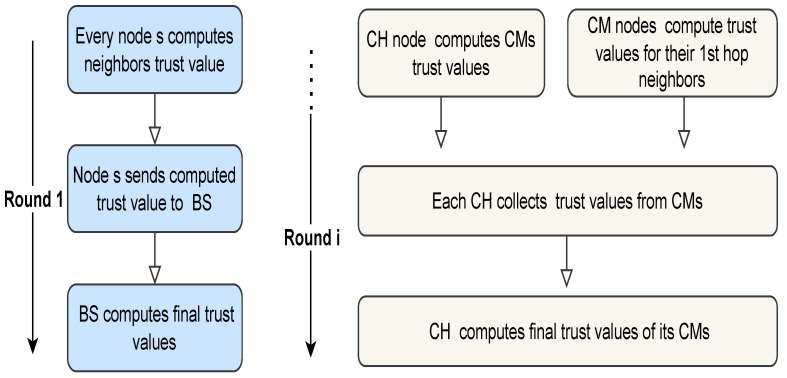
Trust model execution steps [36].

This section explains how the trust value of each SN in the network is determined. It is based on the work in [36]. The different steps executed in the trust model during the first round and the subsequent rounds are shown in Figure 2. As evident from Figure 2, the setup process for trust value calculations starts in the first round (Round 1). In this process, each node monitors its neighbor nodes’ behavior and calculates its neighbors’ trust values based on specific metrics that are determined and adjusted according to the required application. Then, each node sends the calculated trust values to the BS. Following that, the BS divides the nodes into clusters. For the process in the subsequent rounds (Round i), the nodes have been divided into CHs and CMs. Each CM computes the trust value about its neighbors in the same cluster and sends the computed value to its CH. Additionally, the CH computes the trust value of each CM. Then, each CH collects the trust values from its CMs and computes the final trust value of its CMs.

Each SN’s trust value is computed in two tiers on the basis of the trust model. The node tier is the first tier where nodes receive SN and CH trust values. Then, the trust value is determined as the second layer (i.e., the BS tier).

- Trust computation at node tier: The SNs monitor their neighbors’ behavior using a variety of trust metrics. The priority of each metric is determined by the weights allocated to each trust metric (the sum of the weights being equal to 1). According to Equation (3), SN *j* determines the first-hand trust for SN *i*.
  (3) $$FH\left(j,i\right)=\sum_{k=1}^{ł}w_{k}*t_{k}\left(j,i\right)$$
  where *l* represents the total number of trust metrics or parameters, $w_{k}$ denotes the weight associated with metric *k*, $t_{k}\left(j,i\right)$ denotes the trust value set on metric *k* by SN *j* for SN *i*, and
  (4) $$\sum_{k=1}^{ł}w_{k}=1$$
  The respective CH receives the calculated first-hand trust from each SN. The CH aggregates the trust results and determines the aggregated value of trust for each CM in its cluster based on the formula below:
  (5) $$CT\left(i\right)=\frac{1}{b}*\sum_{r=1}^{b}FH\left(r,i\right)$$
  where *b* refers to the count of SN *i*’s neighbors, and FH(r,i) represents the direct trust value computed by SN *r* for SN *i*.
  Additionally, CH computes the first-hand trust value of each CM using Equation (3). Finally, the overall trust value (OT) of a CM is determined using the equation below, where $w_{a}$ and $w_{b}$ are the weights, and $w_{a}+w_{b}=1$.
  (6) $$OT\left(i\right)=w_{a}*FH\left(CH,i\right)+w_{b}*CT\left(i\right)$$
- Trust computation at BS tier: The trust values computed by SNs are sent to the BS, which aggregates them using Equation (5). Finally, the BS determines whether an SN is trustworthy based on a user-defined threshold. Additionally, users will assess the accuracy of the received data from each CH at the BS. If the data is accurate, the respective trust values for CH and CMs are increased.

## 4. Data Gathering Technique Based on Trust and Sparrow Search Algorithm

This section provides our proposed DGTTSSA for trust-aware data gathering in WSN. DGTTSSA uses and modifies the SSA for the efficient clustering of WSN.

### 4.1. Proposed DGTTSSA

Clustering can be regarded as an optimization problem because it seeks to identify a solution among all potential solutions. In an optimization problem, the objective function is maximized or minimized to discover the optimum solution for a complex problem.

Meta-heuristic algorithms are one of the promising methods for resolving such optimization issues. SSA is a meta-heuristic method that can be employed to solve the clustering problems in a WSN. SSA [63] was designed in 2020 to simulate the social behavior of sparrows. In real-world applications, SSA can be used to solve various problems [64]. The SSA population is split into producer and scrounger groups with respect to the foraging behavior of sparrows. Food sources are identified by the producer group, whereas the scrounger group is involved in collecting the food from the producer group. When the sparrow identifies a predator, it generates an alarm, and whenever the alarm value surpasses the threshold for safety, the producers must guide all scroungers toward a safe zone. In the event of danger, the sparrows at the edge of the group fly quickly toward the safe area, whereas those in the center of the group move randomly to be close to one another. The details about the SSA framework can be found in [63,64].

The DGTTSSA clustering is based on SSA and the trust model provided in Section 3.4.

DGTTSSA includes two processes: CH election and Cluster creation. The clustering process is restarted after a period of time if one or more CHs exhaust their energy and the energy level falls below a user-defined threshold.

The DGTTSSA clustering process can be summarized as follows:

1. The BS starts the CH selection operation by broadcasting a request message to each node asking it to send its ID and neighbor list along with its estimated trust value and energy level.
2. The BS determines the final trust value based on the trust values that are gathered from each node.
3. The required number of CHs (*k*) is determined by the BS and is updated during network operations by considering the current count of alive nodes *l* and the required percentage *p* of CHs, where k=l×p.
4. The candidate’s information list is generated by the BS, which includes the node ID, remaining energy, final trust value, and neighbors count.
5. The BS calculates the threshold of residual energy $E_{th}$ using the Equation (8), and calculates the trust threshold $T_{th}$ using the Equation (9).
6. Using the DGTTSSA clustering algorithm, the BS selects the set of trusted nodes to work as CHs in the network.
7. After the selection operation of the CHs, the nearest nodes from each of the elected CHs (that are not CHs) are chosen by the BS to be the CMs for that CH.
8. Nodes designated as CHs receive a message informing them of their selection, while the non-CH nodes receive a message informing them of their CHs.
9. The data collection operation is started by CMs, where CMs deliver the sensed data to their respective CH regularly. Each CH aggregates and transmits the aggregated data to the BS. For the data accuracy review process, the CH includes its ID and each CM’s ID along with the sensed data during the data collection operation.
10. The clustering process is restarted by the BS after a period of time if one or more CHs exhaust their energy and the energy level falls below the threshold. Following steps two to seven, each CH submits relevant information about its cluster to the BS so that the BS can conduct the election process and select the new CHs based on that information.

The subsequent sections provide the different procedures that are employed in DGTTSSA. First, we present the formulation of the fitness function for the DGTTSSA clustering process. Following that, the threshold calculations, initialization procedure, and fix sparrow procedure are explained. Finally, we discuss the working of the proposed DGTTSSA clustering algorithm in detail.

#### 4.1.1. DGTTSSA Fitness Function

CHs in DGTTSSA are chosen based on two factors: (i) trust value and (ii) residual energy. The lowest fitness value is regarded to be the best solution, meaning that we are optimizing in order to minimize the fitness function values. The proposed fitness function is given below.

(7) $$F=\left(exp\left(-\sum_{i=1}^{k}RE_{i}\right)*\frac{1}{CR+1}+exp\left(-\sum_{i=1}^{k}TR_{i}\right)*\frac{1}{CT+1}\right)*\frac{1}{CRT+1}.$$

where CR is defined as the count of CHs that have an energy equal to or higher than the residual energy (RE) threshold. CT is defined as the count of alive CHs that have a trust value equal to or higher than the trust threshold. CRT is defined as the count of CHs that satisfy both of the thresholds. TR represents the trust value, $RE_{i}$ represents the RE of node *i*, and *k* is the CH count.

The fitness function is designed to evaluate the SSA solutions during the CH election process by considering the remaining energy and trust value factors. One of the following four cases could represent the solution:

- CHs have energies below the energy threshold and the trust values are below the trust threshold.
- CHs have energies that satisfy the energy threshold and the trust values are below the trust threshold.
- CHs have energies below the energy threshold and the trust values satisfy the trust threshold.
- CHs have energies that satisfy the energy threshold and the trust values satisfy the trust threshold.

To correctly evaluate these cases, the energies and trust values of the CHs in a solution are weighted by the number of sensor nodes that satisfy each threshold and satisfy both of the thresholds. Since the lowest fitness value is regarded to be the best solution, case 1 will have the largest fitness value and case 4 will have the lowest value.

#### 4.1.2. Threshold Calculation

Equations (8) and (9) provide a tiny decrease in the trust threshold over time compared to the decrease in the energy threshold. The goal of these equations is to form dynamic thresholds that are used in each iteration in order to adapt to the changes in the network during round operations.

The trust and energy thresholds are given by the following formula:

(8) $$E_{TH}=ETH_{max}-\frac{ETH_{max}}{t_{max}}\times \left(t+1\right)$$

where $E_{TH}$ is the energy threshold, $ETH_{max}$ is the maximum threshold value (we use $ETH_{max}=0.05$), the maximum iterations count is $t_{max}$, and the current iteration is *t*.

(9) $$T_{TH}=TTH_{max}-\frac{a\times TTH_{max}}{t_{max}}\times t$$

where $T_{TH}$ is the trust threshold, a∈[0,1] is a small constant (we use $a=1-(E_{current}/E_{Total})$), $TTH_{max}$ is the maximum threshold value (we use $TTH_{max}=0.50$), and $E_{current}$ and $E_{Total}$ are the current and initial total energy values, respectively.

### 4.2. Initialization Procedure

SSA uses a random initializer that is not aware of trust and energy. Therefore, instead of relying solely on SSA’s random initializer that is ignorant of trust and energy, we use an Initialization Procedure (IP) that is aware of trust and energy, and generates a range of solutions for initializing the population. In our procedure, a random value rn∈[0,1] is assigned to each SN in WSN. If the assigned value rn≥T, that node is considered a non-CH node, where *T* is defined by the Equation (10). Otherwise, the node has the chance to be elected as a CH. Algorithm 1 describes the Initialization Procedure. Using this procedure, the population size (Ps) can be adjusted to initialize a part of the population; we set 40% of the population as using the initialization procedure and the remaining as using the random initializer of SSA.

(10) $$T\left(n\right)=\frac{p\times TR_{i}}{\left(1-p\times \left(rmod\left(p^{-1}\right)\right)\right)}\times \left(E_{ratio}\right)$$

where *p* denotes the desired percentage of CHs, *r* represents the sparrow number, $\left[E_{ratio}=\frac{E_{i}}{E_{init}}\right]$ is the energy ratio, $E_{i}$ and $E_{init}$ denote the current and initial energy of node, respectively, and $TR_{i}$ is the final trust value.
**Algorithm 1** Initialization Procedure.1:**Input**: Size of the population Ps, *p* denotes the desired percentage of CHs, Number of sensor nodes n, List of candidate’s information (e.g., Node ID, Energy, and Trust)2:**Output**: Initialized population3:**for** r=1:Ps **do**4:   Initialize $x_{r}$ as zeros {Initialize position of sparrows as zeros}5:   tleft = mod(r,1/p);6:   **for** i=1:n **do**7:     **if** S(i).rleft >0 **then**8:        S(i).rleft = S(i).rleft-1;9:     **end if**10:     **if** S(i).E>0 and S(i).rleft == 0 **then**11:        Generate a random number rn.12:        Compute the value of threshold T(n) based on Equation (10)13:        **if** T(n)≥rn **then**14:           x(r,i)=1 likely to be CH15:          S(i).rleft = 1/p-tleft;16:        **else**17:          x(r,i)=(0.5)×rand(1,1)18:        **end if**19:     **end if**20:   **end for**21:**end for**22:**Return** *x*(Population)

### 4.3. Fix Sparrow Procedure

This procedure is to fix each sparrow *S* to satisfy the boundary conditions such that for each v∈S, v∈[lb,ub], and to have the correct number of required CHs *k*, where ub is the upper boundary and lb is the lower boundary, respectively. The fix sparrow procedure is described in Algorithm 2. In Steps 3–5, correcting the sparrow values that go beyond the boundary is done by assigning the values rand(0,1)×(ub−lb)+lb to each value that goes beyond the boundary. In Steps 7–12, reset the CHs count in a sparrow to preserve only the required *k* CHs.
**Algorithm 2** Fix sparrow procedure.1:**Input**: sparrow X, required *k* CHs2:**Output**: sparrow X containing *k* number of 1’s3:**if** value v in X goes beyond the boundary lb, ub **then**4:   rest v to rand(0,1)×(ub−lb)+lb5:**end if**6:*c* = the number of values that greater than 0.5 in X.7:**if** *c* > *k* **then**8:   Randomly reset c−k values that are greater than 0.5 in X to a random value ∈[0,0.5]9:**end if**10:**if** *c* < *k* **then**11:   Randomly reset k−c values that are less than or equal 0.5 in X to 1.12:**end if**13:**Return** X

### 4.4. DGTTSSA Clustering Algorithm

**Figure 3:**
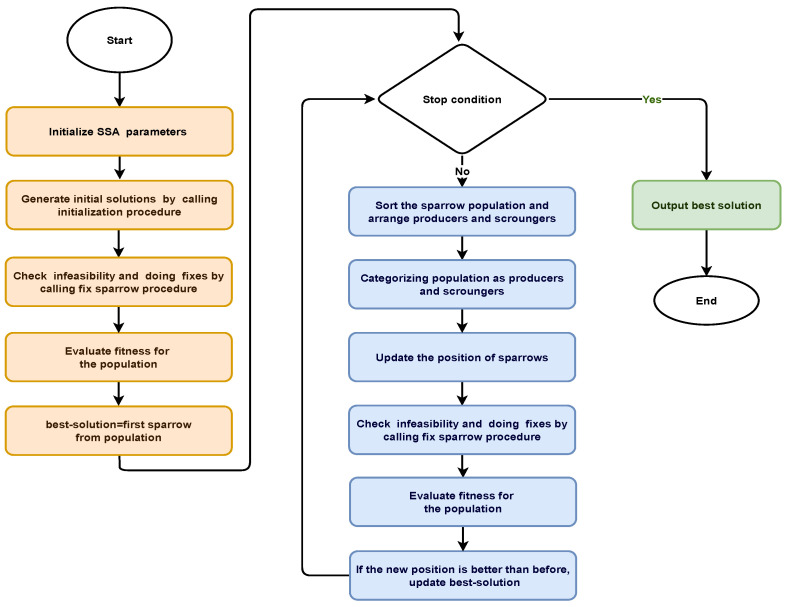
DGTTSSA clustering flow diagram.

This section discusses the proposed DGTTSSA clustering algorithm. In addition to maximizing the WSN lifetime, the algorithm seeks to choose the most trustworthy nodes to serve as CHs. For SSA, a real-valued representation of each sparrow is considered, where the upper boundary equals one and the lower bound is zero, i.e., each value in a sparrow ∈[0,1]. The index denotes node ID and the CH is denoted by a value that is greater than 0.5. On the other hand, non-CH nodes are represented by a value that is less than or equal to 0.5. SSA is adapted in the DGTTSSA clustering algorithm for CH selection, and the fitness function given by Equation (7) is employed to evaluate each generated solution. The lowest fitness value of a solution indicates that this solution is the best one. The flowchart of the DGTTSSA clustering is shown in Figure 3.

The DGTTSSA clustering algorithm is provided in Algorithm 3. The steps involved are given as follows:

1. Initially, the required parameters are set: the percentage of CHs (*p*), the SNs count (*n*), the remaining energy threshold ($E_{th}$, given by Equation (8)), the trust threshold ($T_{th}$ given by Equation (9)), and the maximum iterations (*T*). Set the SSA parameters that include the maximum iterations’ count, number of sparrows (n), producers’ count, danger sparrows’ count, alarm value, and threshold for safety.
2. Initialize the sparrow positions to create a search space randomly and by calling the initialization procedure (Algorithm 1).
3. Check the generated solutions for infeasibility and fix accordingly by calling the fix sparrow procedure (Algorithm 2).
4. Using Equation (7), compute the fitness values for each solution.
5. Obtain the best sparrow solution (best_solution) and the corresponding fitness value (best_fitness).
6. Repeat the following steps until *T* is reached. (Algorithm 3, steps 8–34)
7. Organize the population into producers and scroungers based on each sparrow’s fitness value.
8. Update the position of sparrows using Equations (11)–(13) as follows:
  (a) Updating producer’s position (${x_{i,j}}^{t+1}$) as follows:
    (11) $${x_{i,j}}^{t+1}=\left\{\begin{array}{cc} {x_{i,j}}^{t}\times exp\left(\frac{-i}{\alpha \times T_{max}}\right) & ,\;\mathrm{if}\;R2\;<\;\mathrm{ST}\\ {x_{i,j}}^{t}+Q\times L & ,\;\mathrm{otherwise} \end{array}\right.$$
    where ${x_{i,j}}^{t}$ denotes the $i^{th}$ sparrow position in the *j*th dimension, *t* is the current iteration, and the maximum iteration is $T_{max}$. α∈(0,1] is a random number, R2∈[0,1] is the alert value, ST∈[0.5,1] is the threshold for safety, and *Q* is a normal-distributed number.
  (b) Updating position of the scroungers (${x_{i,j}}^{t+1}$): The rest of the population are scroungers, and their positions are updated as follows:
    (12) $${x_{i,j}}^{t+1}=\left\{\begin{array}{cc} Q\times exp(\frac{{x_{worst}}^{t}-{x_{i,j}}^{t}}{i^{2}}) & ,\;\mathrm{if}\;i>n/2\\ {x_{P}}^{t+1}+\left|{x_{i,j}}^{t}-{x_{P}}^{t+1}\right|\times A^{+}\times L & ,\;\mathrm{otherwise} \end{array}\right.$$
    where $x_{p}$ refers to the producer’s best position, and the worst position is $x_{worst}$. $A^{+}=A^{T}{\left(AA^{T}\right)}^{-1}$ is a 1×d matrix.
  (c) Following the position update of the population, several sparrows are chosen to serve as scouts who identify and alert the rest of the population. They usually constitute 10–20% of the entire sparrow population. Accordingly, the position update equation is given as follows:
    (13) $${x_{i,j}}^{t+1}=\{\begin{array}{c} x_{best}^{t}+\beta \cdot \left|x_{i,j}^{t}-x_{best}^{t}\right|\;,\mathrm{if}\;f_{i}>f_{g}\\ x_{i,j}^{t}+K\cdot \left(\frac{|x_{i,j}^{t}-x_{worst}^{t}|}{(f_{i}-f_{w})+\epsilon }\right)\;,\mathrm{if}\;f_{i}=f_{g} \end{array}$$
    where $x_{best}$ gives the current global optimal position, and the random number β follows a normal distribution with the mean = 0 and variance = 1. $f_{g}$, $f_{w}$, and $f_{i}$ are the global best, worst, and current fitness, respectively; the random number K∈[0,1] and ϵ>0 is a constant. When $f_{i}>f_{g}$, sparrows located at the border of the population are more vulnerable to attacks, whereas the sparrows located at the middle of the population are more likely to avoid predators by staying close to their neighbors when $f_{i}=f_{g}$.
9. Check the generated solutions for infeasibility and do fixes by calling the fix sparrow procedure (Algorithm 2).
10. Using Equation (7), compute the fitness values for each solution.
11. Obtain the best solution for the current iteration (Algorithm 3, steps 27–32).
12. Update the best solution best_solution and its fitness value best_fitness if the current solution is better.
13. Go to step 6.
14. Finally, the best-selected CHs are returned (best_solution and best_fitness).

**Algorithm 3** DGTTSSA clustering algorithm.1:**Input**: SSA parameters, network parameters.2:**Output**: best_fitness and best_solution.3:Initialize the sparrow population position randomly and using initialization procedure (IP) {Each sparrow represents a solution} (Algorithm 1)4:Fix infeasible solutions using the fix sparrow procedure (Algorithm 2).5:Evaluate population using fitness function Equation (7).6:Set bestSolution = individual’s best position7:Set bestFitness = individual’s best fitness8:**while** t≤T **do**9:   Arrange the sparrows into producers and scroungers according to their fitness value.10:   {Update sparrows position}11:   Set r2 to a random number.12:   **for** *i* = 1 : pd **do**13:     Position update for producers using Equation (11).14:   **end for**15:   Fix infeasible solutions using the fix sparrow procedure (Algorithm 2).16:   Evaluate population using fitness function Equation (7).17:   **for** *i* = (pd + 1) : n **do**18:     Position update for scroungers using Equation (12).19:   **end for**20:   Fix infeasible solutions using the fix sparrow procedure (Algorithm 2).21:   Evaluate population using fitness function Equation (7).22:   **for** *l* = 1 : sd **do**23:     Position update for the sparrows in danger using Equation (13).24:   **end for**25:   Fix infeasible solutions using the fix sparrow procedure (Algorithm 2).26:   Evaluate population using fitness function Equation (7).27:  {Get and Update the best solution}28:   Get the best sparrow (CurrecntBestSolution) and the best sparrow’s fitness (CurrentBestFitness)29:   **if** CurrentBestFitness is better than bestFitness **then**30:       Set bestSolution = CurrecntBestSolution31:       Set bestFitness = CurrentBestFitness32:   **end if**33:   *t* = *t* + 134:**end while**35:Return best_solution and best_fitness.

### 4.5. Complexity Analysis

In this section, we provide the complexity analysis of DGTTSSA in terms of communication complexity and time complexity using the following Lemmas.

**Lemma** **1.** *O(n×t×p) is the overall time complexity of DGTTSSA.*

**Proof.**  In order to determine the running cost and performance of the algorithm, time complexity is evaluated. SSA is utilized in DGTTSSA for CH selection. If we assume the SSA population size *p*, the dimension *n*, and maximum iterations *t*, then O(p×t×n) is the time complexity of the SSA [65,66]. In addition to SSA, DGTTSSA includes a feasibility check and fix step for the generated sparrows. As a result, the DGTTSSA algorithm has O(t×(p×n+p×n)) = O(p×t×n) as the overall time complexity. In the data collection process, nodes transmit data to their respective CHs and the CHs send data to the BS. The time complexity is O(k×(n−k)) for the data collection process, where (n−k) is the CM count and k<n is the CH count. Therefore, O(n×t×p) is the overall time complexity of the proposed DGTTSSA. □

**Lemma** **2.** *O(n) is the total communication complexity of DGTTSSA.*

**Proof.** 

- First, *n* nodes send their information to the BS, which means the total communication complexity is *n*.
- Nodes did not transmit any messages in steps 2–8.
- As a result of step 9, *k* CHs are elected and n−k nodes operate as CMs without sending messages.
- Therefore, n+k+(n−k) is the total messages for steps 1–7.
- When time *t* has passed or if one or more CHs have energy levels lower than the defined threshold, the BS initiates steps 2–8 to elect new CHs.
- In the best case scenario, steps 2–8 are executed only once and the total number of messages will be 2n.
- In the worst case scenario, steps 2–8 are executed per round and the total number of messages will be 2n×r, where r is the number of rounds.
- Therefore, O(n) is the total communication complexity of DGTTSSA.

□

## 5. Simulation Results

MATLAB R2016a is utilized as the simulation platform to evaluate the performance of the DGTTSSA approach. The trust value of each node is calculated in the range [0,1], and the malicious nodes percentage is set to 10%. Node energy values are defined in heterogeneous settings, where 10% of the nodes are advanced nodes with initial energy 1 J and the remaining nodes are set as normal nodes with an initial energy of 0.5 J. The system model is previously presented in Section 3, and different network topologies are created randomly for evaluation. An amount of 100 nodes are scattered randomly in the area of size 100×100 m${}^{2}$ with the BS at the center (50, 50), corner (100, 100), and outside (150, 150). We consider 10% as the initial percent of CHs. Table 2 contains the simulation settings. With the aforementioned settings, we compare the performance of the proposed DGTTSSA approach with that of the existing algorithms: ETCHS [57], eeTMFOGA [37], LEACH-TM [56], and E-LEACH [34], with the trust threshold set to 0.5 and energy threshold set to 0.05, respectively.

The following are the performance metrics used for evaluation:

1. Stability Period (SP): it is defined as the time required for the first node to die [67]; i.e., $SP=T_{0}-T_{FND}$, where $T_{0}$ is the time at which the network starts operation and $T_{FND}$ is the time at which the first node dies.
2. Instability Period (InP): it is defined as $InP=T_{FND}-T_{LND}$, where $T_{FND}$ is the time at which the first node dies and $T_{LND}$ is the time at which the last node dies.
3. Network Lifetime: number of nodes that have enough energy to continue their operations.
4. Average residual energy ($ER_{avg}$) per round: the average remaining energy of nodes per round.
5. Reliability (R): this metric is defined by: R=SP/InP [67]. A larger value for *R* indicates good reliability.
6. CHs’ Average Trust Value (ATV): this metric is defined by $ATV=\frac{1}{r_{last}}\sum_{i=1}^{r_{last}}T_{avg}\left(r\right)$, where *r* is the round number, $T_{avg}=\frac{1}{k}\sum_{i=1}^{k}T(i)$, T(i) is the trust value of CH *i*, k is the CHs count, and $r_{last}$ is the round number at which network operations end.

### 5.1. Performance Results of Stability Period and Network Lifetime

**Figure 4:**
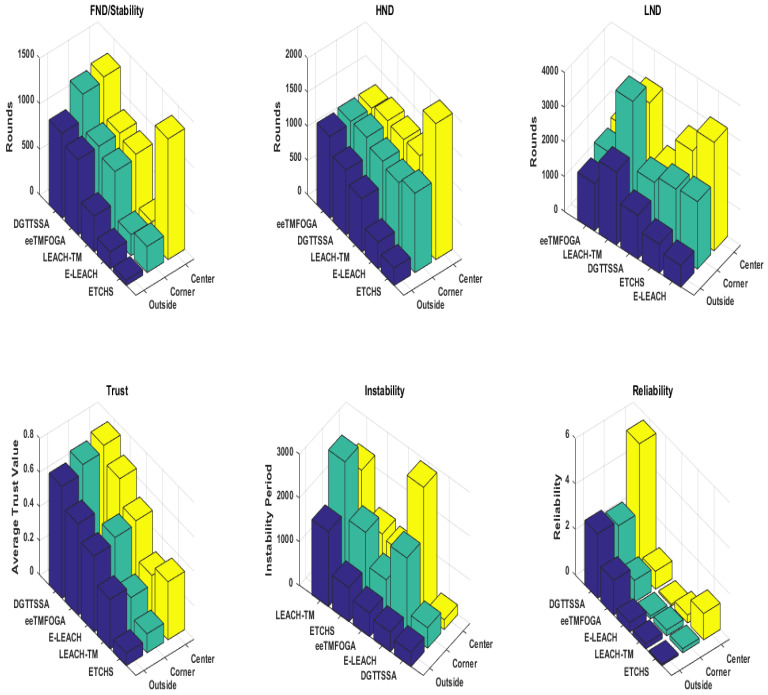
Performance results with reference to various BS locations.

Here, we analyze the stability period of each algorithm with the BS at the corner, center, and outside. Figure 4 shows that DGTTSSA’s stability period is significantly longer than ETCHS, eeTMFOGA, LEACH-TM, and E-LEACH in the case when the BS is at the corner and outside. When the BS is at the center, the DGTTSSA’s stability period is shorter than ETCHS by 5%. It can also be noticed that as the BS position changed from inside to outside the network, ETCHS gives the worst stability period. On the other hand, DGTTSSA’s stability period is slightly decreased. Additionally, the results show that the stability period is longer for all algorithms when the BS is at the center when compared to that when the BS is outside the network. This is because of the increased distance from the nodes to the BS.

**Figure 5:**
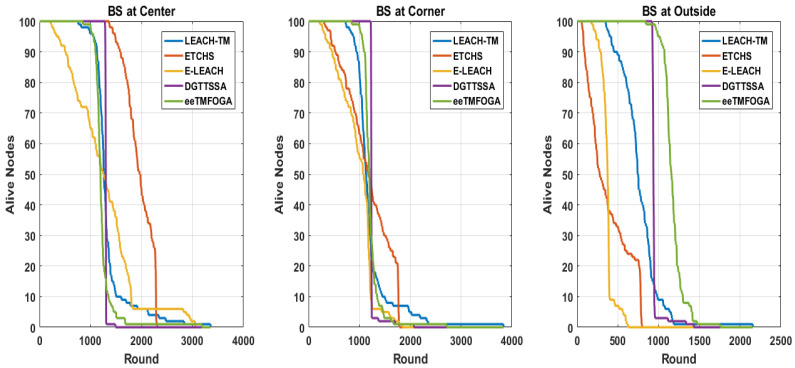
Alive nodes comparison over rounds with reference to different BS locations.

One of the main goals of clustering is to increase the WSN lifetime. According to the application requirements, the WSN lifetime can be considered in terms of first node dies (FND), half node dies (HND), or the last node dies (LND). Figure 4 shows the FND, HND, and LND of DGTTSSA, ETCHS, eeTMFOGA, LEACH-TM, and E-LEACH for different BS location scenarios. Figure 5 shows the alive nodes count per round of operation. It is clear that DGTTSSA has a good performance in terms of FND and HND for different BS location scenarios. E-LEACH has the worst performance in terms of FND, HND, and LND when the BS location is outside because E-LEACH doesn’t consider energy as a selection factor for the CHs.

### 5.2. Performance Results of Energy Consumption

**Figure 6:**
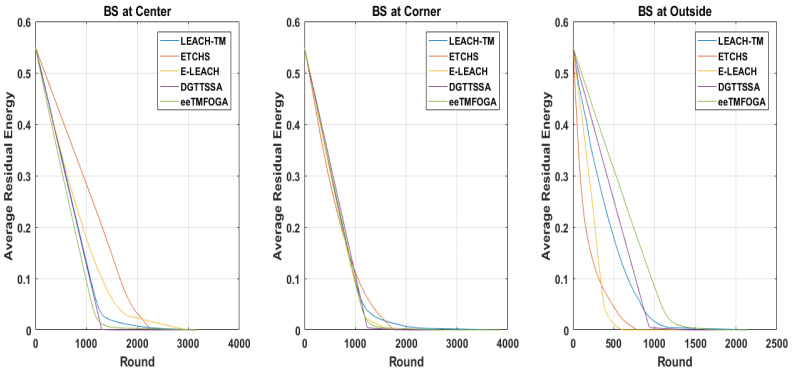
$ER_{avg}$ with reference to different BS locations.

In this section, the energy consumption of the network is evaluated for different BS locations. The RE can be used to estimate how network energy is efficiently used. The average remaining energy in WSN is given by $ER_{avg}=\frac{1}{n}*\sum_{i=1}^{n}RE_{i}$, where *n* is the total nodes count and $RE_{i}$ is the remaining energy of a node *i*. Figure 6 shows the $ER_{avg}$ over rounds when the location of the BS is at the center, corner, and outside. When the BS is positioned at the corner and outside, $ER_{avg}$ consumption is better in DGTTSSA while ETCHS and E-LEACH are better when the BS is at the center and worse when the BS is at the corner and outside.

### 5.3. Performance Results of Reliability and Instability

A comparison of the reliability of different algorithms is given in this section with reference to different BS locations. Figure 4 shows the reliability of DGTTSSA, ETCHS, eeTMFOGA, LEACH-TM, and E-LEACH. It is clear that DGTTSSA has an excellent reliability performance for the different BS location scenarios, whereas the least reliability results are for E-LEACH and LEACH-TM. It is also clear that when the BS is at the center, the reliability is higher when compared to the corner and outside BS locations. Minimizing the instability period is always preferable. DGTTSSA improved the instability period when the BS is at the center compared to LEACH-TM, ETCHS, eeTMFGA, and E-LEACH up to 90%, 80%, 79%, and 92%, respectively. When BS is at the corner, up to 84%, 71%,47%, 73% improvement can be noticed. When BS is located outside, the improvement is up to 81%, 58%, 39%, and 25%, respectively.

### 5.4. Performance Results of CHs Average Trust Value (ATV)

The target of DGTTSSA is to ensure CHs are trustworthy, and this is gained by designing and using a fitness function that selects CHs whose trust values exceed or equals the trust threshold. Figure 4 shows the average trust value for DGTTSSA, ETCHS, eeTMFOGA, LEACH-TM, and E-LEACH. From this figure, it is clear that the ATV of LEACH-TM, ETCHS, and E-LEACH is below the trust threshold value. DGTTSSA gives the best ATV and this is because the fitness function in DGTTSSA assigns more weight to the solutions with CHs that have trust values larger than the threshold value. ETCHS has the worst ATV for the BS at the outside and corner settings, while for the BS at the center scenario, LEACH-TM has the worst ATV.

### 5.5. Performance Results When Varying the Number of Nodes in WSN

**Figure 7:**
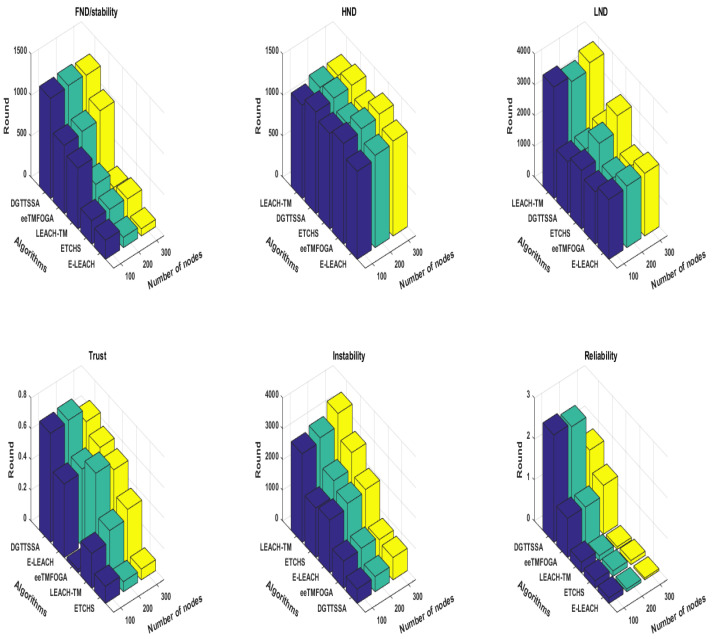
Performance results of varying the number of nodes from 100 to 300 incremented by 100.

Here, we analyze the impact of increasing the number of nodes in the network on the performance of each algorithm. In this test, the trust value of each node is calculated in the range [0,1], and the malicious nodes percentage is set to 10%. Node energy values are defined in heterogeneous settings, where 10% of the nodes are advanced nodes with initial energy 1 J and the remaining nodes are set as normal nodes with an initial energy of 0.5 J. The BS is placed at the corner and the number of nodes varied from 100, 200, and 300. Figure 7 shows the stability period influence with a varying number of nodes. From this figure, it is noticed that DGTTSSA has a stability period in all cases of varying numbers of nodes. Moreover, DGTTSSA still has good stability, ATV, and reliability compared with ETCHS, eeTMFOGA, LEACH-TM, and E-LEACH as the number of nodes increased. It is noticed that the stability period of LEACH-TM and E-LEACH has a significant decrease as the number of nodes increased which is due to both of them employing a probability-based function for CHs selection.

### 5.6. Performance Results When Varying Percentage of Malicious Nodes

**Figure 8:**
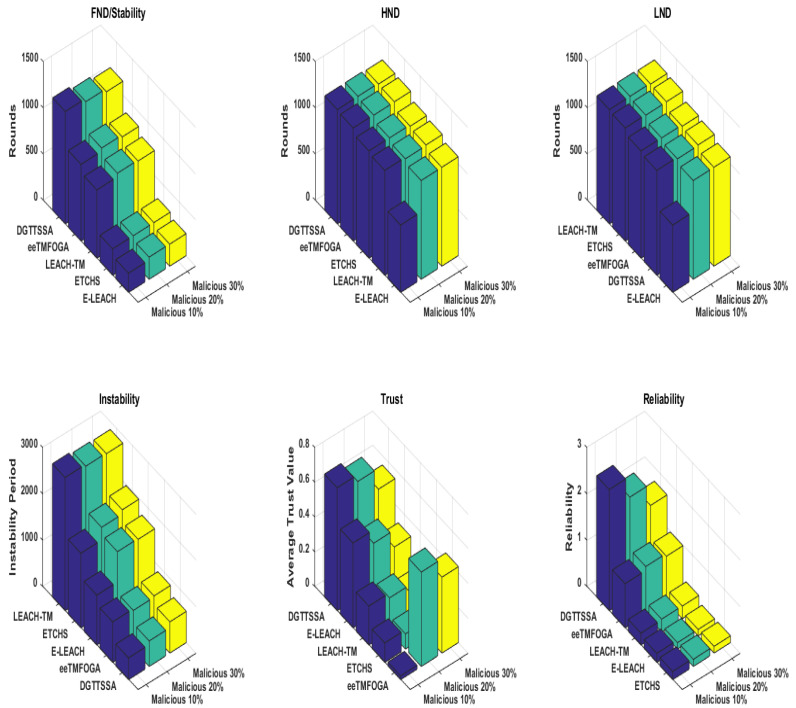
Performance results of varying the percentage of malicious nodes.

**Figure 9:**
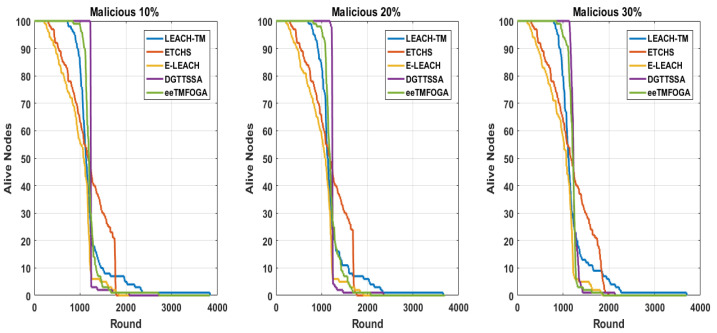
Alive nodes comparison over rounds results of varying the percentage of malicious nodes.

**Figure 10:**
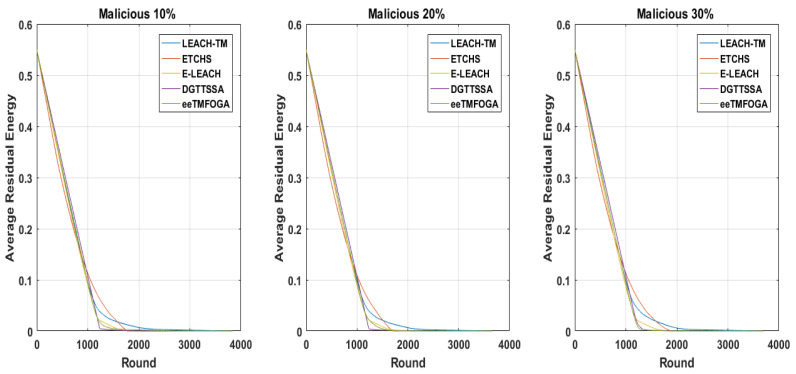
$ER_{avg}$ with reference to varying percentage of malicious nodes.

Here, we discuss the effect of increasing the percentage of malicious nodes (PoMNs) on DGTTSSA, ETCHS, eeTMFOGA, LEACH-TM, and E-LEACH. The PoMNs is varied up to 10%, 20%, and 30% (with BS at the corner) using the same settings presented in Table 2. Figure 8 shows the stability period influence with varying PoMNs values. From this figure, it is noticed that DGTTSSA’s stability period decreases as the PoMNs increases. Moreover, DGTTSSA has better stability than ETCHS, eeTMFOGA, LEACH-TM, and E-LEACH. We can also notice from Figure 8 that the instability period for DGTTSSA and ETCHS increased as the PoMNs increased. Moreover, it is clear that LEACH-TM has the highest instability period followed by ETCHS, E-LEACH, eeTMFOGA, and then DGTTSSA. The relationship between alive nodes’ count over time and the PoMNs is shown in Figure 9. It can be noticed from this figure that when PoMNs is 30%, the nodes die earlier. The effect of the PoMNs on reliability, as given by Figure 8, shows that the reliability is dropped in DGTTSSA as PoMNs is increased. In all PoMNs values, the DGTTSSA has the best performance compared to the others. Moreover, ETCHS and E-LEACH have the least reliability for all PoMNs values. The effect of PoMNs on the ATV illustrated in Figure 8 shows that the ATV value decreases as PoMNs increases. This is because the choices available for electing nodes with a high energy and trust value are decreased; despite this, DGTTSSA picks the CHs whose ATV values are greater than the trust threshold value for all PoMNs values. This is due to the fact that the DGTTSSA’s design considers both energy and trust when evaluating and choosing nodes. Additionally, it can be noticed from Figure 8 that ETCHS and LEACH-TM provide the least ATV for all PoMNs values. From Figure 10, it can be observed that the average residual energy decreases as the PoMNs increases and follows the same pattern.

In summary, Table 3 shows a comparison of the proposed DGTTSSA and other algorithms in terms of FND, HND, LND, ATV, stability, instability, and reliability for different BS locations. Table 4 shows a comparison of the proposed DGTTSSA with others in terms of FND, HND, LND, ATV, stability, instability, and reliability for 10%, 20%, and 30% of malicious nodes. Hard thresholds for trust and energy employed in ETCHS for CH selection have a negative effect on the network lifetime, reliability, and ATV. While LEACH-TM and E-LEACH algorithms adopt a random CH selection mechanism which may increase the probability of electing nodes with low trust values, LEACH-TM employs the trust aware random mechanism and E-LEACH employs a random mechanism that is not trust-aware. Generally, random mechanisms may increase instability periods and reduce reliability and the ATV. Since eeTMFOGA has no threshold for trust values, CHs with lower trust values may be selected. In addition, when the PoMNs increases, it is obvious that nodes with lower trust values are selected. On the other hand, DGTTSSA adapts SSA and is modified by initializing its population with a partially generated population based on a trust-aware random mechanism and with a fitness function designed to be weighted by nodes’ counts that satisfy both energy and trust thresholds. Moreover, DGTTSSA employs dynamic energy and trust thresholds to overcome hard threshold drawbacks that do not reflect the current network state. The benefit of this is an improvement in network lifetime, stability, reliability, and ATV.

## 6. Conclusions

In this paper, a data gathering technique that employs trust and the Sparrow Search Algorithm for WSN (DGTTSSA) is introduced. In DGTTSSA, the SSA is utilized and modified for secure CH selection. Our fitness function takes two parameters into account: residual energy and trust value, to yield trustworthy and energy-efficient CHs. The modified SSA is used for determining a set of nodes that have better residual energy and trust values to serve as CHs. We employ dynamic threshold values for energy and trust which are adjusted according to the network state. The simulation results show that the DGTTSSA is reliable, more stable, and provides a longer lifetime for the WSN than other methods. In addition, under varying percentages of malicious nodes, DGTTSSA selects the most trustworthy nodes as CHs. As a result, for applications based on WSNs that require safe and efficient clustering, DGTTSSA can be used as a secure and efficacious clustering solution to enable efficient data gathering.

## Figures and Tables

**Table 1:** Related works comparison.

Ref.	Trust Aware Clustering	Approach	Limitations
MG-LEACH (2019) [33]	×	Nodes are divided into subgroups and CHs are selected randomly based on the probability function. Data is routed to sink through intermediate CH.	CH selection based on the probability function can lead to early depletion of nodes. Trustworthiness of nodes is not considered.
E-LEACH (2019) [34]	×	CH selection is based on the probability function and the shortest distance is employed for CH identification.	Nodes’ residual energy is not considered. CH selection based on probability function can lead to early depletion of nodes. Nodes’ trustworthiness is not considered.
RCH-LEACH (2020) [35]	×	CH selection is based on nodes’ residual energy along with the energy threshold and best number of clusters.	CH selection based on probability function can lead to early depletion of nodes. Hard energy threshold and trustworthiness of nodes are not considered.
eeTMFOGA (2020) [37]	√	Hybrid algorithm based on moth flame optimization and genetic algorithm with a fitness function that considers average cluster distance, energy, trust, and node density.	Missing threshold on trust value may lead to the selection of CHs with a lower trust value.
HiTSeC (2018) [36]	√	Bat Optimization Algorithm is employed for CH selection. A hard trust threshold is considered for CH selection. The objective function is designed based on the degree of the node.	Hard threshold is employed. Uses a weak fitness function.
LEACH-TM (2021) [56]	√	LEACH and trust based algorithm. Trust value of nodes are considered in a randomized selection function.	CH selection based on probability function can lead to early depletion of nodes and leads to the selection of CHs with a lower trust value.
ETOR [39] (2021)	√	Employ intra-cluster and inter-cluster multi-hop communication. Fitness function is based on energy, trust, QoS, and connectivity between route nodes.	Complex method. Missing threshold on trust value may lead to the selection of CHs with a lower trust value.
TAGA [41] (2022)	√	CH selection is based on a randomized function that considers comprehensive trust value and remaining energy.	CH selection based on randomized function can lead to early depletion of nodes and leads to the selection of CHs with a lower trust value.
[46] (2021)	√	Cuckoo search optimization using a fuzzy type-2 logic is used for clustering.	Hard threshold is employed.
[47] (2022)	√	CH selection based on hybrid optimization for optimal result by considering energy, separation distance, delay, distance, Qos, and trust.	Missing trust threshold may lead to the selection of CHs with a lower trust value.
[49] (2022)	√	Employs Deep learning and Whale Optimization based method for CH selection, using a multi-objective function considering the distance, energy, delay, and trust of nodes.	Complex method and missing threshold on trust value may lead to the selection of CHs with a lower trust value.
[50] (2021)	√	CH selection based on PSO with a fitness function that considers factors such as energy, delay, trust, the consistency factor, and the maintainability factor.	Missing threshold on trust value may lead to the selection of CHs with a lower trust value.
STELR [51] (2022)	×	Emperor penguin optimization (EPO) is employed for CH selection while considering distance and energy.	Selection of CHs with a lower trust value.
TE-MHOA [53] (2022)	√	Hybrid algorithm consists of adaptive Particle Swarm Optimization and Monarch Butterfly Optimization with a fitness function considering residual energy, communication cost, trust, and node degree.	Missing threshold on trust value may lead to the selection of CHs with a lower trust value.
[54] (2021)	×	Employs Randomized function and genetic algorithm for CH selection.	CH selection based on probability function can lead to early depletion of nodes and nodes’ trustworthiness is not considered.
ETCH [57] (2019)	√	CH is selected by considering the trust value and residual energy.	Hard threshold is employed. Uses a weak fitness function.
TC-MBWO [59] (2022)	√	CH selection is achieved using a trust-aware method based on Multi-objective Black Widow Optimization.	Missing threshold on trust value may lead to the selection of CHs with a lower trust value.
Proposed DGTTSSA	√	The energy and Trust aware fitness function is designed along with the soft energy and trust threshold calculation method. SSA is employed for CH selection.	QoS parameters not considered.

√ means “considered” and × means “not considered”.

**Table 2:** Simulation Settings.

Used Parameters	Value
Area	100×100 m
Total nodes (n)	100
Initial node energy	normal nodes = 0.5 J; advanced nodes = 1 J
$E_{elec}$	50 nJ/bit
Free space $\epsilon_{fs}$	10 pJ/bit/m${}^{2}$
Multi-path $\epsilon_{m}p$	0.00013 pJ/bit/m${}^{4}$
$d_{0}$	87 m
$E_{DA}$	5 nJ/bit/signal
Packet size	4000 bits
Communication radius	40 m
Percentage of CHs (p)	0.1
Range of trust value	[0, 1]
**Parameters used for SSA**	
Population size	30
Percentage of producers	0.2
Percent of sparrows in danger	0.2
Safety threshold	0.8
Max iterations	100

**Table 3:** Results when BS is at Center, Corner, and Outside.

BS Is at Center						
	**Stability/FND**	**ATV**	**Instability**	**Reliability**	**HND**	**LND**
LEACH-TM	786.40	0.29	2256.40	0.35	1264.4	3042.8
DGTTSSA	1264.40	**0.65**	**224.00**	**5.64**	1291.6	1488.4
ETCHS	**1325.00**	0.07	1146.40	1.16	**1990**	2471.4
eeTMFOGA	833.00	0.53	1061.00	0.79	1228.4	1894
E-LEACH	229.20	0.44	2895.20	0.08	1270.8	**3124.4**
**BS Is at Corner**						
	**Stability/FND**	**ATV**	**Instability**	**Reliability**	**HND**	**LND**
LEACH-TM	739.00	0.22	2873.60	0.26	1135.6	**3612.6**
DGTTSSA	**1217.20**	**0.71**	**465.60**	**2.61**	**1237.8**	1682.8
ETCHS	286.40	0.11	1609.60	0.18	1161.6	1896
eeTMFOGA	830.80	0.022	871.00	0.95	1224.4	1701.8
E-LEACH	231.80	0.48	1710.60	0.14	1070.6	1942.4
**BS Is at Outside**						
	**Stability/FND**	**ATV**	**Instability**	**Reliability**	**HND**	**LND**
LEACH-TM	390.60	0.28	1708.40	0.23	776.2	**2099**
DGTTSSA	**930.60**	**0.74**	**329.40**	**2.83**	948.8	1260
ETCHS	57.80	0.34	777.60	0.07	255.8	835.4
eeTMFOGA	825.60	0.65	541.20	1.53	**1193.6**	1366.8
E-LEACH	184.60	0.50	442.00	0.42	387.4	626.6

Bold indicates the best value for this column.

**Table 4:** Results with varying percentage of malicious nodes (10%, 20%, and 30%).

10% Malicious Nodes						
	**Stability/FND**	**ATV**	**Instability**	**Reliability**	**HND**	**LND**
LEACH-TM	739.00	0.22	2873.60	0.26	1135.6	**3612.6**
DGTTSSA	**1217.20**	**0.71**	**465.60**	**2.61**	**1237.8**	1682.8
ETCHS	286.40	0.11	1609.60	0.18	1161.6	1896
eeTMFOGA	830.80	0.54	871.00	0.95	1224.4	1701.8
E-LEACH	208.20	0.49	1076.30	0.19	729	1284.5
**20% Malicious Nodes**						
	**Stability/FND**	**ATV**	**Instability**	**Reliability**	**HND**	**LND**
LEACH-TM	778.00	0.20	2831.60	0.27	1118.8	**3609.6**
DGTTSSA	**1181.60**	**0.67**	**545.60**	**2.17**	**1239.4**	1727.2
ETCHS	286.40	0.09	1884.40	0.15	1159.8	2170.8
eeTMFOGA	865.00	0.54	832.80	1.04	1220.6	1697.8
E-LEACH	240.00	0.41	1729.60	0.14	1069	1969.6
**30% Malicious Nodes**						
	**Stability/FND**	**ATV**	**Instability**	**Reliability**	**HND**	**LND**
LEACH-TM	767.60	0.15	2817.60	0.27	1126.4	**3585.2**
DGTTSSA	**1146.60**	**0.55**	**673.60**	**1.70**	**1230**	1820.2
ETCHS	286.40	0.05	1978.60	0.14	1144.4	2265
eeTMFOGA	843.00	0.44	862.20	0.98	1223.6	1705.2
E-LEACH	238.60	0.31	1712.00	0.14	1070.4	1950.6

Bold indicates the best value for this column.

## Data Availability

Not applicable.
